# An Efficient Dynamic-Decision Based Task Scheduler for Task Offloading Optimization and Energy Management in Mobile Cloud Computing

**DOI:** 10.3390/s21134527

**Published:** 2021-07-01

**Authors:** Abid Ali, Muhammad Munawar Iqbal, Harun Jamil, Faiza Qayyum, Sohail Jabbar, Omar Cheikhrouhou, Mohammed Baz, Faisal Jamil

**Affiliations:** 1 Department of Computer Science, University of Engineering and Technology, Taxila 47080, Pakistan; abidali.hzr@gmail.com (A.A.); munwariq@gmail.com (M.M.I.); 2 Department of Electronic Engineering, Jeju National University, Jeju 63243, Korea; harunjamil@hotmail.com; 3 Department of Computer Engineering, Jeju National University, Jeju 63243, Korea; faizaqayyum@jejunu.ac.kr; 4 Department of Computational Sciences, The University of Faisalabad, Faisalabad 38000, Pakistan; sjabbar.research@gmail.com; 5 CES Laboratory, National School of Engineers of Sfax, University of Sfax, Sfax 3038, Tunisia; omar.cheikhrouhou@isetsf.rnu.tn; 6 Department of Computer Engineering, College of Computer and Information Technology, Taif University, P.O. Box 11099, Taif 21994, Saudi Arabia; mo.baz@tu.edu.sa

**Keywords:** mobile cloud computing, fault tolerance, task scheduling, offloading, cloud virtual machines

## Abstract

Restricted abilities of mobile devices in terms of storage, computation, time, energy supply, and transmission causes issues related to energy optimization and time management while processing tasks on mobile phones. This issue pertains to multifarious mobile device-related dimensions, including mobile cloud computing, fog computing, and edge computing. On the contrary, mobile devices’ dearth of storage and processing power originates several issues for optimal energy and time management. These problems intensify the process of task retaining and offloading on mobile devices. This paper presents a novel task scheduling algorithm that addresses energy consumption and time execution by proposing an energy-efficient dynamic decision-based method. The proposed model quickly adapts to the cloud computing tasks and energy and time computation of mobile devices. Furthermore, we present a novel task scheduling server that performs the offloading computation process on the cloud, enhancing the mobile device’s decision-making ability and computational performance during task offloading. The process of task scheduling harnesses the proposed empirical algorithm. The outcomes of this study enable effective task scheduling wherein energy consumption and task scheduling reduces significantly.

## 1. Introduction

Advancements in the Information and Communication Technologies sector and related Internet of Things (IoT) technologies have an impact on humans’ lives all over the world. IoT is, in simple words, intelligent things equipped with sensors that gather data without the interactions of humans. These devices are smart enough to collect/capture, handle, transmit and display the resulted data without any manual automation. The IoT concept provides the capability to connect almost everything around us that can communicate using the internet. The objective is to connect mobile devices to promote their task processing capabilities. Mobile cloud computing is a combination of IoT devices such as smartphones that use IoT-based technology to enhance the task processing capabilities of mobile devices.

In recent years, computing and information technology resources have been increased and deployed in many fields such as cloud, mobile cloud, fog computing, distributed, and parallel computing. In all these technological advancements, there is a rapid increase in the development and use of cloud computing. Ultimately, different resources are needed to manage and handle mobile devices to cloud plate-forms [1,2]. Mobile cloud computing (MCC) is the systematical use of the cloud plate-form for smaller mobile devices and other smaller machines to provide connectivity in terms of the network for sharing data, different mobile operating systems, processing capacity, apps, storage, etc. [3]. The cloud itself is a massive data center available 24/7 to provide different homogeneous and heterogeneous resources to millions of connected mobile users for fast and efficient access to resources at their locations worldwide [4]. Before we proceed with the MCC’s actual challenging area problems, the problem is understanding MCC’s study with a few definitions from different literature reviews. MCC uses applications that are running on a remote server with immense processing capabilities. The enormous storage and mobile devices need different formats to serve mobile users [5]. MCC comprises different resource-rich servers, mobile devices, network devices, a cloud operating system, and local and remote applications. These are connected and maintained in a decidedly organized manner. The resources must be handled, maintained, and efficiently shared across the cloud devices [6].

Today, due to the multiple benefits of the cloud, the world is shifting towards MCC. MCC’s main research areas include processing power, battery lifetime, encrypted copying, and transferring the different mobile applications from the cloud to MCC users [7]. Efficient task submission and processing for fault tolerance, resource sharing, reuse of computing resources over multiple devices, or even the same computing device to allow internet-based applications like games, online simulation techniques, etc. MCC can leverage the existing challenges of mobile devices and shift them to MCC [8].

However, it is notable that Cloud Computing (CC) and MCC are not new technologies that provide services to mobile users. MCC is a form of CC. CC provides the data management operations on cloud virtual machine Virtual Machines (VM). Simultaneously, the MCC integrates CC expertise with mobile devices to make the mobile devices resourceful in expressions of energy, computational power, storage, memory, and context awareness. Virtualization and utility-based pricing use different cloud services in a more refined manner [9,10,11]. At the MCC Foundation, several devices connect with the cloud to access or offer various cloud services to the users and other third-party cloud platforms [12].

Multiple mobile devices want to access cloud services at a remote location or cloud servers. Initially, the request is directed to the local cloud, which is local to the mobile network provider (MNP). With a database and central server working in coordination with the local mobile cloud network (MCN), it prepares the request to deliver via the internet for cloud services. If the requested resource is not found, it asks to be redirected to the remote cloud where all the services are offered and available for processing [13]. Moreover, MCC helps mobile devices to save energy and processing time by offloading certain resources and computationally intensive tasks to the mobile cloud. The studies found that large tasks can be broken down into smaller tasks and task precedence requirements. The fine granularity of the task level offloading can achieve both time and energy saving [14].

Furthermore, task scheduling is optimized using MCC, offloading it from a mobile device towards the MCC VM. One of the more suitable strategies for deploying MCC is to offload all computations to the computational cloud for processing and deliver the results back to the mobile cloud service. Different cloud service providers (CSP) provide such services to their cloud users for better understanding and convenience [15]. Several existing methodologies such as intelligent batteries, power scheduling, efficient operating system, energy-aware communication protocols, and applications are introduced to reduce mobile devices’ energy and time consumption. So, task offloading is introduced to reduce the requirements of these mobile device tasks. Task Scheduling helps the mobile devices to reduce the battery life and improve the handling of mobile device energy and time optimizations. The task offloading helps overcome the communication time cost and energy of these devices to overcome such challenges. Thus, the task that should be offloaded from mobile devices to the MCC VM must be decided based on some significant factors like memory, execution time, processing energy, network bandwidth, processor utilization, allocation time, upload time, results in processing time, and some local executions parameters. Thus, task scheduling is one of the main fields to consider in MCC research objectives. Besides all this, every mobile user can experience these services at any time without any time limitations and interruptions [16]. However, user data, applications, confidential information, and locations are insecure for cloud users due to compromised attacks in this field [17,18].

Computation-intensive tasks (tasks sizes are larger than computational capabilities) are rich in computations. Energy and time consumption are the two main factors to manage in mobile cloud computing. Energy and time are the challenges faced during task scheduling in mobile cloud computing. Limited capabilities of mobile devices in terms of energy, storage, time, transmission bandwidth, and computations trigger problems associated with energy optimization and time management while processing tasks on a mobile phone. This problem needs to be addressed in this paper, focusing on efficient dynamic decision-based task scheduling in MCC.

The main contribution of the proposed system is to save time and computational energy using the mobile cloud computing approach as follows:

- The main objective of this system is dynamic decision-making for task scheduling using the decision-based algorithm.
- The task offloading decision is straightforward, using a dynamic decision-based scheduler to predict which task is offloaded to the mobile cloud and which task is executed on the mobile device.
- The controller effectively decides to enhance the efficiency of the decision algorithm by making choices in less time.
- The decision algorithm works collectively with the scheduler to enhance the probability of task-processing decision-making.
- We effectively reduce the power consumed by mobile devices’ task execution through task scheduling decision algorithms and task competition models.
- Finally, for evaluation of the system performance, we analyze the results using mobile offloading through simulation. Our proposed technique indicates that the decision algorithm effectively improves the system decision-making, and less power is consumed through dynamic decision-making for task execution.

The rest of the paper is organized as follows. Section 2 presents the related work on task scheduling and fault tolerance. Then, in Section 3, we outline our approach for the research and proposed solutions to the relevant problems encountered in Section 1 of the paper. Then, in Section 4, we present the proposed method with simulations using a hybrid approach. In Section 5, we conclude our proposed system, highlighting that our technique makes it straightforward for fault tolerance methodology.

## 2. Related Work

In MCC, several studies have recently been discussed in the literature. Zhuo et al. [19] proposed a group-based fault tolerance technique called GFT-mCloud, which can classify mobile devices into different groups based on reliability, processing capacity, and mobility. Based on the task offloading schemes, specific groups of jobs are scheduled based on the criteria. Noraziah et al. [20] implemented a fault tolerance technique in which cloud providers and cloud users/customers share their responsibilities in response to fault tolerance techniques to efficiently utilize resources and energy efficiency. As in [21,22], different application faults can be repaired and detected at the user level, however, hardware and virtual machine faults can be identified and repaired at the cloud level. The recovery/restoration of the applications running on the refurbished VMs can be requested and performed at the customer level. The checkpointing technique is used to create re-establish points for the recovered VMs.

Chen et al. [23] proposed a new method for concentering fault tolerance in a cloud. They used a k-out-of-n approach that mobile devices can successfully retrieve and process different data in a very effective energy-efficient way as remote servers are accessible for processing. Park et al. [24] developed a new technique based on the Markov Chain Model system, based on the analysis and prediction of the status of all resources available. Li et al. [25] discovered that clouds are more resistant to the fault problems caused by mobile devices’ mobility. Abd et al. [26] defined the cloud computing models that support on-demand Internet access, convenient, ubiquitous access to different bundles of resources for configurable computing. It has gained an enormous amount of popularity. The National Institute of Standards and Technology (NIST) has defined that cloud computing has many distinguishing characteristics for the cloud, such as measured services, on-demand self-service, rapid elasticity, resource pooling, and broad network access. The high distribution of resources and heterogeneity makes the system more convenient and suitable for cloud resource distribution [27]. Fault tolerance is one of the significant issues in cloud computing that needs to be addressed when designing the cloud system for efficiency. Guo et al. [28] analyzed that hundred to millions of clients were affected whenever any cloud resources failed for any reason, which leads to disastrous implications.

Data processing and retrieval, which demonstrates that a big file is split and encoded and then distributed to the network for evaluation [29]. In Table 1, we have compared the recent techniques for MCC task scheduling considering fault rate, makespan, energy optimization, offloading, heterogeneity, control messages, storage, and percentage of tasks executed on either mobile devices or MCC. The table significantly handles the comparison based on different proposed techniques. The table clearly shows that the energy optimization and makespan are not evaluated in MCC in combination.

## 3. Proposed Model

**Figure 1:**
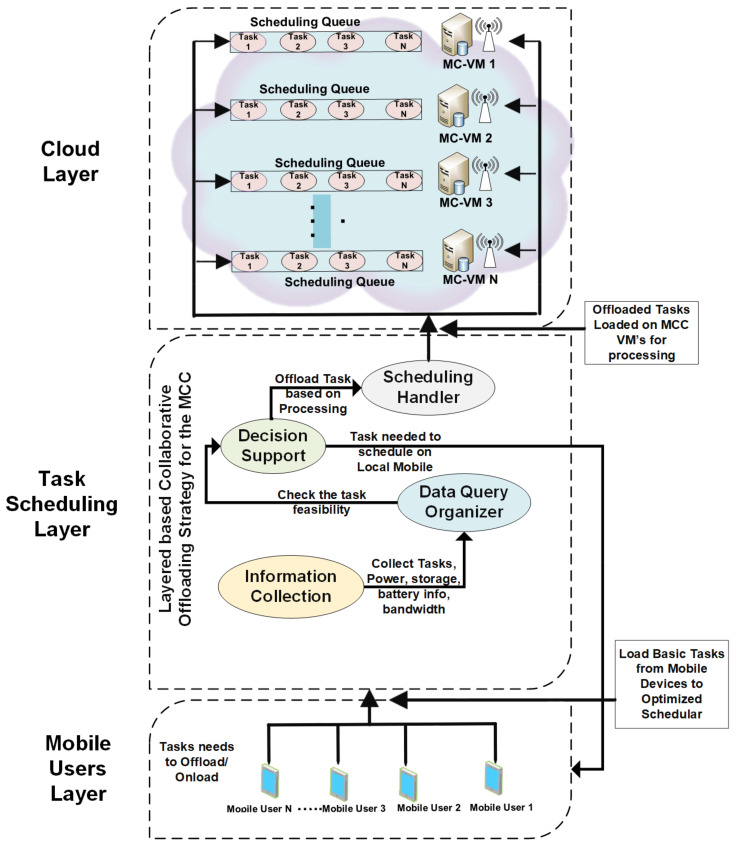
System architecture with task processing and scheduling strategies.

The following section provides a detailed overview of a system architecture proposed and implemented to efficiently handle the mobile cloud fault tolerance using a hybrid approach for access and the cloud services and other main scenarios. Consider the scenario where the tasks are either offloaded to the MCC VM or to processes on a local mobile device. Figure 1 shows the system architecture diagram of the three-layer architecture. The mobile user layer consists of mobile devices with local mobile task processors. The mobile devices are interconnected through high-speed wi-fi or other facilities. The second layer is the task scheduling layer. The task scheduling layer provides task decision capabilities with a highly intelligent scheduling algorithm. All the modules are discussed in the below-mentioned sub-headings. The task scheduling layer’s final task should be processed either on the local machine/mobile device or offloaded to the MCC VM. The decision is based on the computational process and task information gathered through the mobile device. Every task is handled individually. The cloud layer is where the final and updated tasks are received and processed with high-speed processing capabilities. The MCC handler manages and assigns a separate VM to an individual task. These tasks are passed to the task scheduling layer. The main functions of the task scheduling layer are.

(a) Scheduling Handler: Handle multiple application services access and provide a dynamic scheduling technique for managing and distributing numerous services over the cloud.
(b) Information Collection: Collect all information from the mobile devices accessing the services, like power information, processing, storage, battery information, bandwidth, processing capacity.
(c) Information Processing/Checking: This part of the processor checks the above information to assess that the particular cloud service is suitable for handling the different mobile devices or not.
(d) Scheduling Information Keeper: This Information Collaboration Site (ICS) module is important because it keeps the information related to the cloud services and some other information whilst the service is being used by a specific mobile device. After completion, the ICS automatically deletes the information from its storage.
(e) Decision Support (DS): The DS module decides about the processing and other mobile device capacities and decides whether to allocate the cloud services to the mobile or not. The final allocation is based on the decision of this module.
(f) Data Query Organizer (DQO): The DQO is responsible for data inflow and outflow from cloud services based on what type of request is received, and what kind of service needs to be distributed to the mobile client. Besides, to handle the computational costs and the results of the data query returned from the cloud processors.

**Figure 2:**
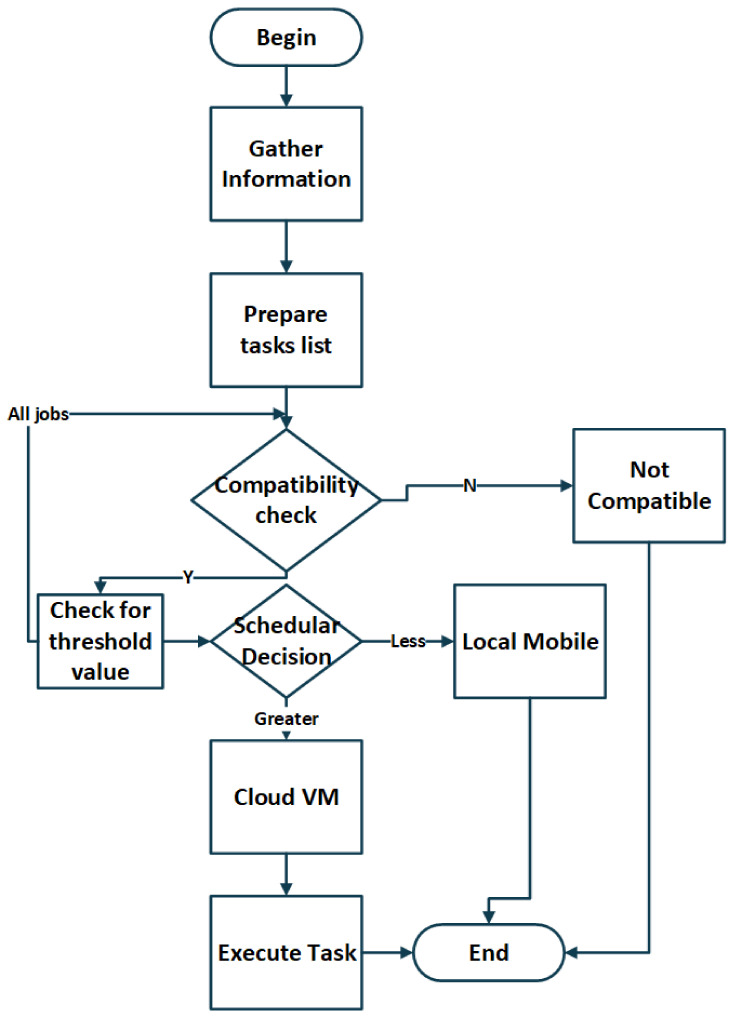
Task Flow Diagram.

**Figure 3:**
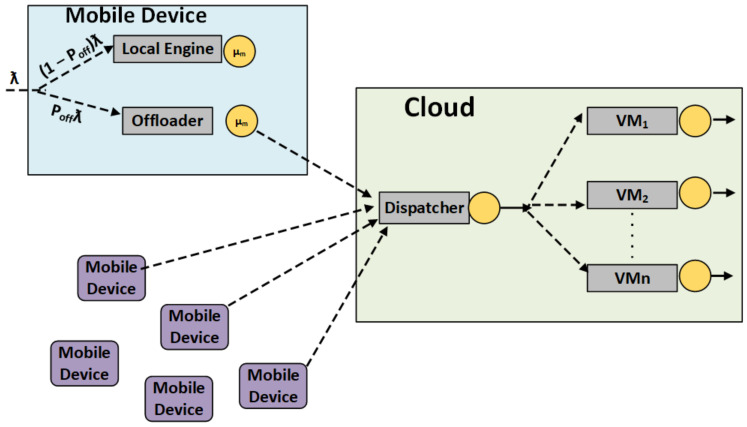
Network Distribution Model for Mobile Cloud System.

Figure 2 and Figure 3 depict the working of the task scheduling layer. All three layers are processed according to their assigned tasks. Initially, the information collection module of Layer 2 receives the mobile devices’ data for strategic decision-making. When the task is not sufficient for the local mobile device computation, the data query organizer organizes and supports this device to process these steps. The task passed from the data query organizer is now well established and ready to decide the task offloading towards the cloud layer. The scheduling handler manages and finalizes the offload to the mobile cloud layer. The complete strategy is a layer-based collaborative offloading strategy to offload the tasks to the mobile cloud layer.

In our proposed system, whole scheduling and fault tolerance methods are handled at the middle layer. The decision is made at this layer before the request has reached the cloud for processing and handling. Figure 2 shows the system model diagram, which consists of the flow of tasks from one module of the prosed MCC system to another. Scheduling of the jobs needs to be handled by the scheduler, whereas the jobs need to be submitted to the MCC for processing.

### System Model

Our proposed model constructs two separate networks: task handling and VM networking to process task distribution and task handling. There are two basic networking models, one for mobile devices. The mobile device can process basic applications and enhance the capabilities for classifying the network system, consisting of a mobile device local engine and mobile device off-loader for offloading the tasks/jobs to the cloud for the relevant processing. The equation that describes the local engine to solve the task is (1 − P_off_)λ. The task offloading capability of the mobile devices is checked based on the job’s time for every task. The time calculation probability on the mobile device is calculated with Equation (1). The model represents the local mobile calculation and task submission towards the mobile cloud. (1 − P_off_)λ represents the time subtracted from the total mobile calculated time.

ΔT_total mobile_ = ΔT_m_ + ΔT_e_ − ΔT_ec_ − ΔT_on_ − (1 − ΔT_off_) (1)

In Equation (1), ΔT_total mobile_ calculates the total time of the local mobile for calculation and submission for processing. In this equation, the λ represents the system’s job request rate that required the mobile to get jobs/tasks from the devices. The ΔT_total mobile_ total time indicates the total time for calculations on mobile devices. Similarly, if it requires a job to be scheduled on the cloud for the dispatcher/scheduler, its time is calculated using

ƩT_off_λ = ΔT_on_ + ΔT_e_ + ΔT_m_ + ΔT_off_ (2)

where ƩT_off_λ is the precise time for task submission until dispatch for scheduling. Furthermore, the scheduler can reject any task based on the task’s information with proper execution and extension. The total time from process load to return after execution on the cloud is calculated with Equation (3). In Equation (3), ƩT_total_λ represents the total time from when the process starts to create and finalize the computed values.

ƩT_total_λ = P_off_(ΔT_off_ + ΔT_ec_) + (1 + ΔT_on_ + ΔT_m_) (3)

We have used offloading based on the FCFS (First Come First Served) and the priority-based hybrid model of the offloading queue for dispatching to the cloud for execution in this model.

Job J is the set of all independent tasks executed on Central Processing Unit (CPU) cores based on N_t_^(j)^. The numbers of tasks collectively form the whole job for execution, and if the mobile contains a single core, then the tasks are done one after another in a sequential manner or if the mobile has multiple CPU cores, N_c_, all tasks are executed in a parallel manner. While on the other hand, on the cloud, all jobs/tasks will be executed in parallel on different cloud virtual machines. To speed up the execution of a job/task, we take the ratio of execution of the tasks and jobs for which it is executed. So,

F = α N_t_^(j)^/N_c_ (4)

where α is the clock frequency ratio for mobile CPU cores and VM processors. The fault rate of any mobile device is estimated using the following information from the mobile device before dispatching it to the cloud. Power is a significant concern for handling the fault rate. To manage power resources, we needed to adapt to the data rate of the mobile device that needed to be handled. For task offloading, we checked the task probability first to determine if the task should be offloaded to the cloud because, for some tasks/jobs, the mobile device can handle them using Equation (5).

(5) $$P_{\mathrm{off}\left(\mathrm{task}\right)}=\left\{\underset{i}{\cup }\lambda (N_{t}{}^{\left(j\right)}+\;C)\right\}<A\left(N_{c}\right)$$

where U is the union of all tasks that group together a job for offloading, λ is the request rate of a mobile needing to execute on the cloud, N_t_^(j)^ is the total number of tasks performed on the cloud, C is the job size (mainly in instructions), A is the clock frequency ratio of the mobile core processor, N_c_. In this model, first, the task is checked to determine whether the mobile CPU core(s) can handle the job. If they can, then the task will be executed on the mobile. Otherwise, it will be offloaded to cloud VM, which is modeled in the next step. Amoretti et al. [19] developed a model for offloading based on the energy consumed to execute by the mobile is higher than the energy consumed by the cloud VM. Still, in our approach, the power will also be wasted due to the massive network consumption power. We checked for fault tolerance based on mobile battery information, location information, and mobile storage information by using the following model.

Equation (6) describes the battery information through the specified threshold.

(6) $$M_{b}\;\geq {\;M}_{\left(b\right)\mathrm{threshold}}$$

Equation (7) represents the mobile location, with no changes to the new low bandwidth network.

M_loc_ (B) ≥ M_new-loc_(B) (7)

In Equation (8), the mobile storage is less than that of the output results produced from the cloud VM to the mobile.

M_storage_ < B + Cloud_output_ (8)

In our research case, we needed to handle the fault tolerance before the task is distributed to the cloud. This helps the dispatcher check all the mobile device’s required details to dispatch to the cloud plate form. Figure 3 shows the execution sequence of task processing from the local mobile device towards the MCC. The estimated Time ΔT_ec_ is reflected from the scheduler.

The task scheduling process based on Algorithm 1 (below), which estimates the job, needs to be executed on mobile and the job performed on the cloud VM. Initially, the algorithm gathers the mobile device information through mob_info(T, B, L, S, App), the number of jobs through jobs_num(m), the number of mobile nodes with nodes_num(n), and gets the mobile information with fetch_info(T). Based on the information gained through steps 1, 2, 3, and 4, new mobile cloud nodes, a job schedular, and cloud VMs are created. The mobile device sends the information to the schedular with arguments. If the execution time is less than the justified threshold value, then the task should be processed on the mobile cloud, and if the task is heavyweight, then the task is uploaded towards the cloud VM. The equation below in step 7 of the cloud estimation plan based on this approach allows the system to continuously check the job execution time computed through Equations (1)–(3). The system then schedules the task for either the cloud VM or local mobile devices. Finally, the algorithm returns the job state.
**Algorithm 1.** Task Scheduling Decision**Input:** Input from Table 1 (LEGENDS Table) **Output:** Returns the state of the job submitted to the cloud or processed on mobile device/decision about mobile or cloud execution
1:mob_info(T, B, L, S, App)2:jobs_num(m)3:nodes_num(n)4:fetch_info(T)5:create_nodes(N, CloudVM, Schedular)6:send(T, D, C)   scheduler( )7:**for** (T ≥ 0) **do**$\;\;\;\;\;\;\;\;\;\;\;calculate_{exe\_time}$( )       F ← $\frac{{\Delta T}_{m}}{{\Delta T}_{\mathrm{exc}}}$ **end for**8:**while** (job_size ≤ threshold) **do**     C(B, F, M_b_, M_loc_, M_storage_)     **if** (C ≤ M) **then**     $job\_exe_{M}$( )     **else**     $active_{Cloud}$(V.M.)     $submit_{C\left(J\right)}$( )     $exe_{job}$( )     **end if****end while**      9:$Job\_state_{Store}$( )

## 4. Simulation Environment

To evaluate the effectiveness of the proposed model for MCC, we implemented a simulation based on a mobile cloud simulator called CloudSIM and a mobile cloud simulator called Cloud Analyst (which is based on CloudSIM with an improved GUI interface). To the best of our knowledge, the CloudSIM is the best simulator for the simulation of Cloud and MCC environments, just like real-world environments. Cloud Analyst, built on top of CloudSIM, is a very active and user-friendly environment for a specific mobile cloud simulation setup. CloudSIM simulator is for mobile simulation on local machines with limited battery, processing (number of CPUs and cores), RAM, Internet and bandwidth, processing time (in milliseconds), etc. First, we simulated the results based on task availability on local machine simulations. If the cloud affected local machines, then the simulator was working fine on the availability of tasks on the local mobile. Based on the task information and mobile capacity information, the simulator would choose the probability and perform all of the local mobile simulations. At first mobile, we evaluated the information that we obtained in the following fashion.

We took ten chosen tasks randomly from the system in the initial simulations and considered the first iteration for simulation for the results extraction. Table 2 shows the information that is extracted from the tasks using a mobile phone. ΔT_total mobile_ is the time calculated using Equation (1) from the above simulations. When using the total time for mobile execution, the system must represent the following calculation as an example. The information is collected after simulation steps and it provides the complete details of the tasks and their processing capabilities. Based on the data captured from the mobile devices, the CM and the local mobile devices are ready to process the data after the scheduler’s decision.

ΔT_total mobile_ = 0.5 + 0.7 − 0 − 0 − (1 − 0) = 1.2 ms

Related to these calculations, we cleared that the first task was evaluated in line with the above calculations. Next in the list, we calculated all the jobs requiring calculation using Table 3.

ƩT_off_λ is the tasks offloading time. For MCC, it is the total time for the task being shifted from the local mobile to the cloud. This time is evaluated by Equation (2) from the model and for the evaluation of offloading time using Equation (2) for task 1 we have

ƩT_off_λ = ΔT_on_ + (ΔT_total mobile_ − ΔT_m_) + ΔT_m_ + ΔT_off_ ƩT_off_λ = 0.012 + (1.2 − 0.5) + 0.5 + 2.31 = 3.342 ms

**Figure 4:**
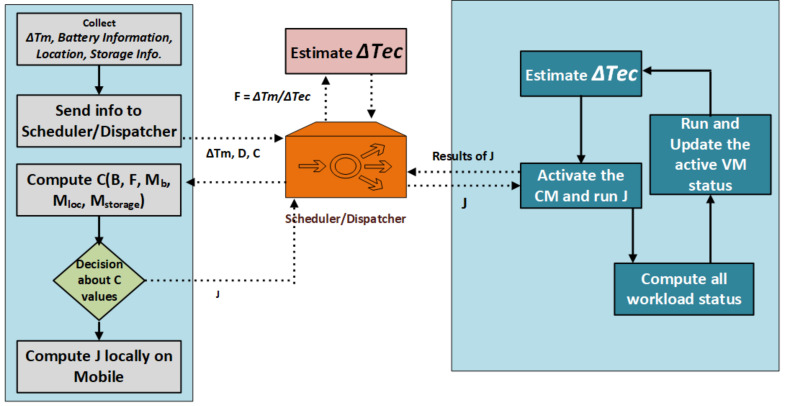
Job execution sequence in MCC.

After calculating all ƩT_off_λ times for the calculations, we simulate and extract the results based on the simulation scenarios. Figure 4 depicts the actual results to get the tasks processed on MCC or local mobile devices.

**Figure 5:**
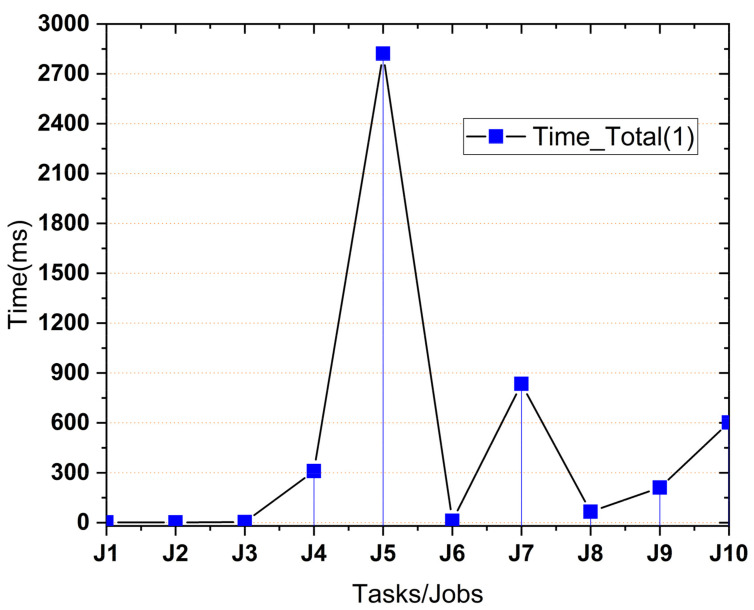
Time calculations on the mobile device.

ƩT_total_λ time is the total time from task creation from the mobile device to the cloud. This time includes the time in which the mobile device decides the offloading or local device calculations. The decision taken by the mobile is that the device is ready to offload. Figure 5 shows the time calculation completed with offloading time and the time required by the cloud VM for execution. From Equation (3), we calculated the total time for a task from the mobile device to the cloud for processing with the formulae below.

ƩT_total_λ = (ΔT_off_ + ΔT_ec_) + (1 + ΔT_on_ + ΔT_m_) ƩT_total_λ = (3.342 + 1.2) + (1 + 0.3 + 0.5) ƩT_total_λ = (4.542) + (1.8) ƩT_total_λ = (6.342) ms

**Figure 6:**
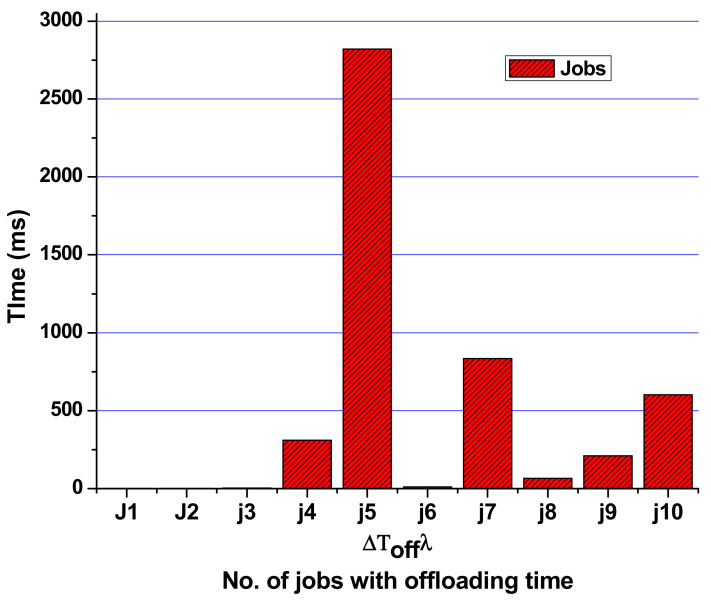
Total offloading time from MCC to the cloud virtual machine (VM).

The total time for a task is used for decision purposes to calculate the task for offload, calculations for the cloud, and calculations for the mobile. Figure 6 shows the percentage of power consumed by the tasks. Some tasks show that higher power consumption means these tasks should be offloaded for MCC, and other tasks should be executed on local mobile devices.

**Figure 7:**
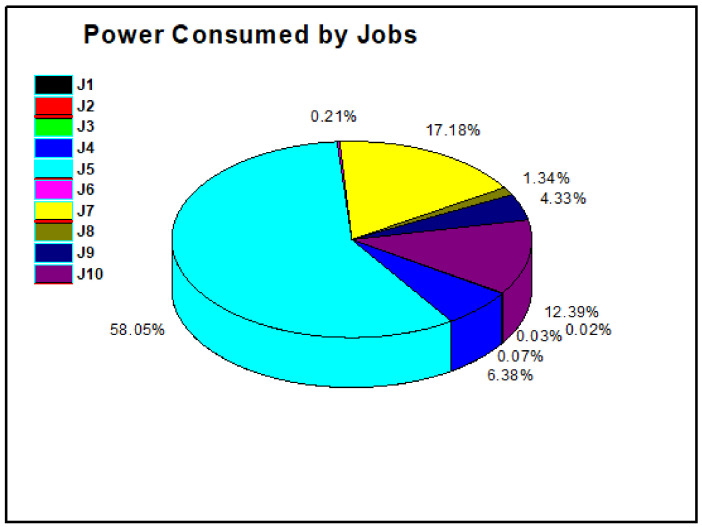
ƩT_total_λ with power P used for the jobs.

**Figure 8:**
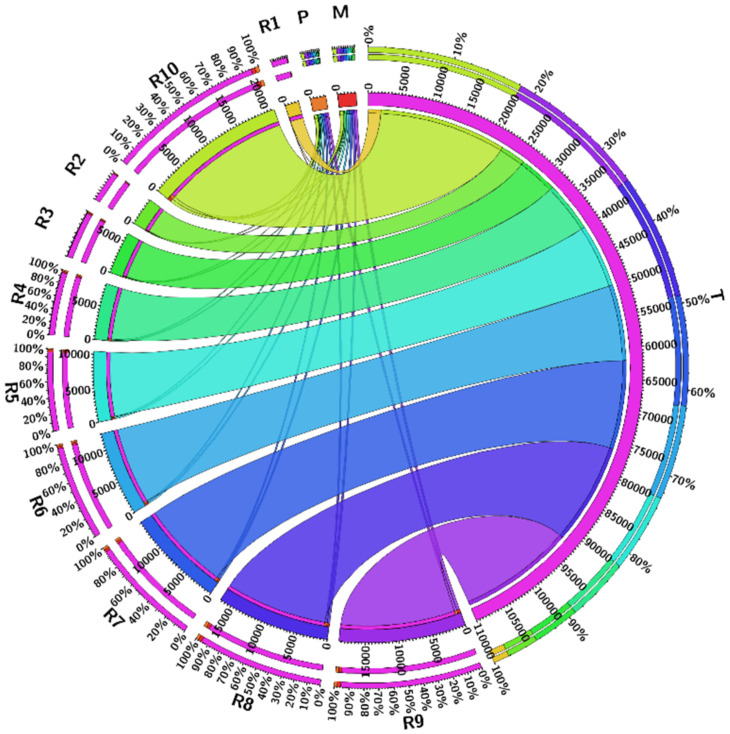
Energy optimization results for the proposed technique in comparison with Mukherjee et al. [22].

Time consumption ƩT_total_λ with power P was used for the tasks at different time intervals during the simulations. Figure 7 shows the comparison of task total time and power used by the task for processing on the mobile and cloud in the proposed system. It is observed that if tasks are increased in batch (number of jobs become batch), the calculations are less, so the power required for the task is less than other techniques. The probability is computed through Equation (8). After the computation of the probability, Figure 8 shows the highest and lowest offloading probability for task processing energy optimization. There are two approaches, P and T. P shows our proposed algorithm, and T shows the existing approach [22]. We analyze the energy of the tasks from 2000 to 20,000 energy efficiency costs for all tasks. Based on the graphs, we conclude that the proposed technique efficiently reduces energy consumption while offloading the tasks. On every single node, all the results effectively handle the tasks offloading and processing energy consumption for mobile and VM processing.

**Figure 9:**
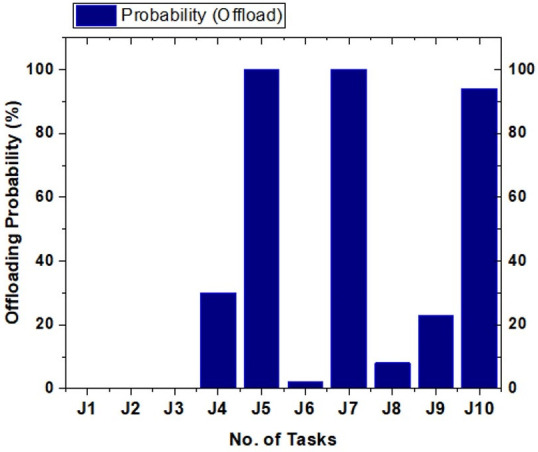
The decision of task offloading probability.

Task offloading time ƩT_off_λ from Equation (2), is the total time we need to offload the responsibility from the mobile to another if it uses the battery power of the mobile machine. So for passing the results of the remaining time, Equation (5) is used with the constraints from Equations (6)–(8). From this, it is observed that only the tasks that have a computation time higher than the required calculations are shifted to the cloud VM for calculations. Figure 9 shows the time and task offloading decisions based on calculations.

**Figure 10:**
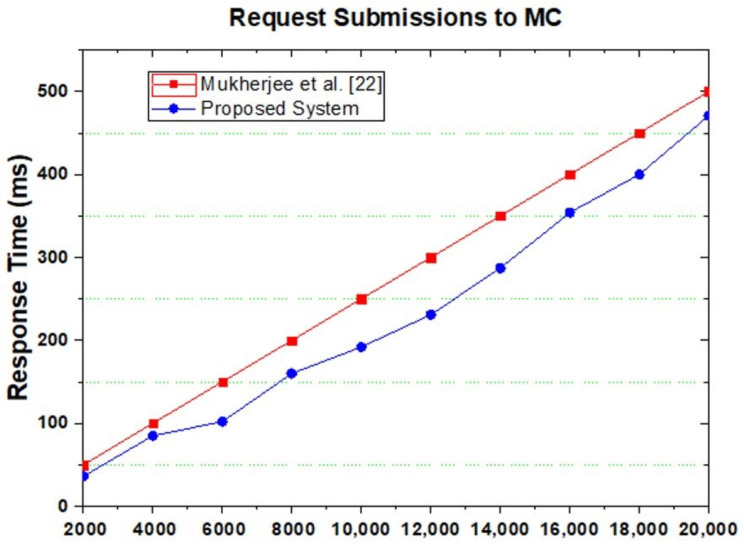
Request submitted to the cloud of Mukherjee et al. [22] and proposed system.

The major decision is based on the task execution time. The load on the cloud is decreased because we do not offload jobs to the cloud VM until it possesses the required probability value, which is set to offload the jobs for processing on the cloud VM. Figure 10 shows the probability of tasks transferred to the cloud VM and demonstrates that the proposed system requires fewer results than the existing system in [22].

**Figure 11:**
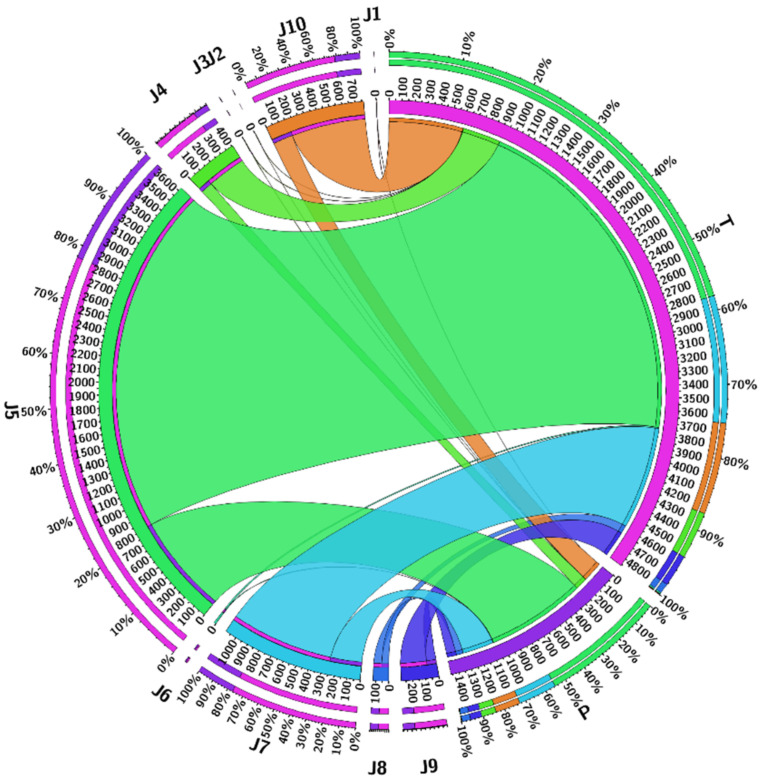
Energy optimization request submitted to the cloud of Mukherjee et al. [22] and proposed system.

For the probability of task offloading and scheduling tasks for transfer to the cloud VM for high processing, Equation (5) is used with the conditions checked through Equations (6)–(8), respectively, after the simulation takes place. The simulation setup based on the results calculates the task scheduling based on the algorithm proposed in the system. The probability and statistics results for the tasks of the mobile device configurations are shown in Figure 11. The graph clearly indicates that task processing energy is optimized from T to P. As its shows that T consumes higher power than P on every job from $\sum_{j=1}^{10}J_{i},$ the proposed approach effectively saves the processing energy from the mobile device for MCC, the consequences of the final task offloading decision are based on the following indicators:

- Battery information
- Bandwidth
- Storage
- Offloading time
- Job completion rate

Table 4 shows the mobile device’s indicators to offload the task based on the statistics of the mobile device to execute tasks, which are related to the various job scenarios. Certain task times are significantly above the specified threshold value. The scheduler will schedule the offload based on these results.

## 5. Conclusions

This paper presents a proposal and simulation relative to MCC and the Cloud VM with a task scheduling technique based on the task time parameter. We explored task scheduling and task enhancement effects on MCC to improve the performance of mobile devices. Based on the proposed model in this paper, we submit a new task scheduling policy that incorporates and provides offloading mobile device calculation and offloading probability based on the time calculation of different scenarios of task scheduling from the mobile device to the cloud. MCC is an essential factor in offloading the tasks, which significantly affects the task handling and task scheduling policies from mobile devices to cloud resources. The technique enables mobile users to effectively balance the time bounds for efficient time handling capabilities. We considered two scenarios; the first involved tasks being executed on mobile devices. The second involved tasks being completed on the cloud VM. Time and energy constraints were used to assess when time efficiency had been achieved, and energy automatically managed. Our proposal scheduled shorter execution time and energy-optimized tasks to mobile devices, with larger and energy-intensive tasks being offloaded to the cloud. Our strategy effectively achieves time efficiency, energy efficiency, better performance, higher resource utilization, and task execution success without diminishing performance quality. Moreover, the results showed that the jobs which go beyond the time thresholds will be shifted to the cloud VM for processing to save the mobile device’s limited battery power.

## 6. Future Work

We will consider a privacy-aware and authentic approach to handle task scheduling. We will determine better task modeling and decision-making capabilities by using blockchain-based privacy-aware task scheduling through MCC as a better and essential research direction. In this regard, the currently applied model will provide better support for this work.

## Figures and Tables

**Table 1:** Comparison of some proposed MCC-related solutions in the literature.

S.No	Proposed Papers	Algorithm Used	Fault Rate	Makespan Time	Energy Optimization	Offloading	Heterogeneity	Control Messages	Storage	% of Task Executed
1	Lee et al. [30]	Group based fault tolerance	✓	✓	-	✓	✓	✓	-	-
2	Raju et al. [31]	Disease Resistance Approach	✓	✓	-	-	✓	-	-	✓
3	Abd et al. [26]	k-out-of-n framework (denoted by KNF)	✓	✓	✓	-	✓	-	-	✓
4	Park et al. [24]	MARKOV chain based monitoring Model	✓	✓	✓	✓	-	✓	✓	✓
5	Al-Sayed et al. [32]	Dynamic Grouping Technique	✓	✓	-	-	-	-	-	-
6	Kashanchi et al. [33]	A genetic method for task scheduling	✓	-	-	✓	-	-	✓	-
7	Peng et al. [34]	Reliability-compliant and Energy-aware Data Storage	✓	-	✓	-	✓	-	✓	-
8	Tang et al. [35]	Energy-Efficient Task Scheduling	✓	-	✓	-	-	-	-	✓
9	Lin et al. [36]	Performance-Aware Task Scheduling	-	-	✓	✓	-	-	-	✓
10	Guo et al. [37]	EETS. Model for Task Scheduling	-	-	✓	✓	-	✓	-	-
11	Wei et al. [38]	MLMCM for Task Scheduling	-	✓	-	-	✓	-	-	-
12	Nawrocki et al. [39]	M L through Adoptive service	-	✓	-	-	-	✓	✓	-
13	Akki et al. [40]	N.N. based optimization methods	-	-	✓	✓	-	-	-	✓
14	Shakarami et. al. [41]	stochastic-based offloading approaches	-	✓	✓	✓	-	✓	-	✓

**Table 2:** List of parameters and variables used in the system.

S.No	Lagend	Description
1	K	Number of cloud virtual machines that are representing the cloud {k_max_, k_min_}
2	J	Job from anywhere on android phone, task request rate (customarily considered as per mobile device)
3	P_off_	Mobile task offloading probability
4	N_t_^(j)^	Number of tasks that forms a job (j)
5	N_c_	The average number of cores of the CPU for the mobile
6	A	Clock frequency ratio
7	B	The bandwidth available to the mobile
8	C	Job size in terms of instructions
9	S_c_	Cloud machine speedup
10	C(B,C,S_c_)	Mobile device energy balance
11	M	Instructions/second (job/task execution speed)
12	D	Data transfer amount (in bytes)
13	R	RAM required on memory (in bytes)
14	W_m_	Average power used by mobile device
15	W_i_	Power used by mobile when idle
16	W_off_	Power used by mobile when it is offloading a job to the cloud VM
17	W_on_	Power used when network enabled on the mobile device
18	E_on_	Energy to turn the network interface
19	ΔT_on_	Average time for turning on the network interface
20	ΔT_e_	Average time for task/job execution
21	ΔT_m_	Average mobile job execution time
22	ΔT_off_	Average time required for the offloading process
23	ΔT_ec_	Average execution time on the cloud
24	ΔT_ret_	Average job return time from cloud VM
25	Ø_tc_	The ratio between waiting and execution time on the mobile or cloud
26	S	Setpoint for Ø_tc_
27	D	Parameter for the adaptive cloud controller
28	ƒ_ct_	Tasks completed on the cloud
29	Q	Probability of tasks at low-parallelism
30	F	Cloud speed-up is estimated using the formula.
31	M_b_	Mobile battery information
32	M_L_	Mobile location information
33	M_store_	Mobile storage information
34	M_(b)threshold_	Estimated battery required for backup for the offload of a task
35	M_loc_	Mobile current location
36	M_new-loc_	New location of the mobile

**Table 3:** Task Data Collected through CloudSIM Simulator.

S.No.	Tasks	ΔT_m_ (ms)	Battery Information (mAh)	Location	M_ore_ (Storage, Mb)	ΔT_total Mobile_ (ms)	B (Bandwidth, Kb/s)	CPU Cores	RAM (Gb)	K_min_, K_max_
1	J_1_	0.5	0.2	33.9944073 72.9335021	5	1.2	131	1	2	5, 20
2	J_2_	0.8	0.4	31.25440053 70.5335021	8	1.4	131	1	2	5, 20
3	J_3_	1.8	0.7	32.2356291 69.7629013	12	3.2	131	1	2	5, 20
4	J_4_	199	1.47	33.9944073 72.9335021	82	310.21	131	1	2	5, 20
5	J_5_	2000.2	15.2	33.9944073 72.9335021	503	2821.4	131	3	2	5, 20
6	J_6_	5.77	12.6	33.9944073 72.9335021	9	10.42	131	1	2	5, 20
7	J_7_	789.45	14.6	33.9944073 72.9335021	392	834.91	131	2	2	5, 20
8	J_8_	43.2	6.8	33.9944073 72.9335021	34	65.23	131	1	2	5, 20
9	J_9_	122	11.5	33.9944073 72.9335021	61	210.41	131	1	2	5, 20
10	J_10_	450.81	28.6	33.9944073 72.9335021	242	602.31	131	2	2	5, 20

**Table 4:** Mobile Task Decision based on the Time from Mobile Device to Cloud.

Tasks	ΔT_total_λ (ns)	Decision	Decision Value (Flag 0/1)
J1	6.342	Mobile	0
J2	8.422	Mobile	0
J3	12.362	Mobile	0
J4	838.482	Cloud	1
J5	7796.932	Cloud	1
J6	29.494	Mobile	0
J7	2498.302	Cloud	1
J8	182.702	Mobile	0
J9	356.802	Mobile	0

## Data Availability

Not applicable.
